# The modified Hardinge approach is not inferior to trochanteric flip osteotomy for Pipkin type IV femoral head fractures: a comparative study in 40 patients

**DOI:** 10.1007/s00068-024-02547-2

**Published:** 2024-05-15

**Authors:** I-Jung Chen, Ying-Chao Chou, Po-Ju Lai, Yung-Heng Hsu, Yi-Hsun Yu

**Affiliations:** 1 Division of Orthopedic Traumatology, Department of Orthopedic Surgery, Chang Gung Memorial Hospital, No. 5, Fu-Hsing Street, Kweishan 333, Taoyuan, Taiwan; 2 Bone and Joint Research Center, Chang Gung Memorial Hospital, No. 5, Fu-Hsing Street, Kweishan 333, Taoyuan, Taiwan

**Keywords:** Femoral head fracture, Acetabular fracture, Hardinge approach, Trochanteric flip osteotomy

## Abstract

**Purpose:**

To compare the modified Hardinge approach and trochanteric flip osteotomy for the treatment of Pipkin type IV femoral head fractures.

**Methods:**

This retrospective study included 40 patients who underwent surgical treatment for Pipkin type IV femoral head fractures between 2011 and 2020 and completed at least 1 year of follow-up. The clinical outcome of the Merle d’Aubigné-Postel score and radiological outcomes, including the quality of the fracture reduction, osteonecrosis of the femoral head, posttraumatic osteoarthritis, and heterotopic ossification, were compared between the two groups. Conversion to total hip replacement was recorded as the main outcome measure, analyzed by Kaplan–Meier curve and log-rank test.

**Results:**

Nineteen and 21 patients were treated using the modified Hardinge approach (Group A) and trochanteric flip osteotomy (Group B), respectively. The estimated surgical blood loss was significantly higher in Group B (500.00 ± 315.44 mL vs. 246.32 ± 141.35 mL; *P* = 0.002). Two patients in Group B complained of discomfort caused by the trochanteric screws and requested implant removal. Radiographic outcomes did not differ significantly between the two groups. Clinical outcomes assessed using the Merle d’Aubigné-Postel score 1 year after injury were nearly identical (*P* = 0.836). Four (21.1%) patients in Group A and three (14.3%) patients in Group B underwent conversion to total hip replacement during the follow-up period; the log-rank test showed no significant difference (*P* = 0.796).

**Conclusions:**

The modified Hardinge approach resulted in reduced blood loss, with clinical and radiological outcomes similar to those of trochanteric osteotomy; thus, it is an acceptable alternative to trochanteric flip osteotomy.

## Introduction

Femoral head (FH) fractures are relatively uncommon injuries mostly caused by high-energy trauma, such as traffic collisions, falls from a height, and industrial mishaps [1–3]. In 1957, Pipkin proposed a simple and useful method to classify FH fractures [4]. Among the four types of fractures described, combined FH and acetabular fractures (Pipkin type IV) are believed to be associated with poorer prognoses [4–8]. The optimal surgical approach for combined lesions remains controversial as addressing both fractures using a single surgical approach is difficult.

Surgical hip dislocation with trochanteric flip osteotomy has been described for the management of femoral-acetabular impingement, acetabular fractures, and FH fractures [9–13]. This technique allows for the simultaneous exposure and repair of both elements of Pipkin type IV fractures without compromising the FH vasculature; [14] however, nonunion, migration of the trochanteric osteotomies, and implant-related trochanteric bursitis have been reported as complications of this approach [9, 15–17].

The use of the modified Hardinge approach to treat Pipkin type IV FH fractures has been previously described [18]. After the soft tissues around the hip joint are dissected, the joint can be dislocated, and the fractured fragments of the FH can be managed without trochanteric osteotomy. The acetabular component can be reduced and repaired simultaneously using a single surgical approach.

The aim of the current study was to compare two surgical approaches, modified Hardinge and trochanteric flip osteotomies, used for the surgical treatment of Pipkin type IV FH fractures. The quality of fracture reduction and radiographic and clinical outcomes were reported. We hypothesized that the modified Hardinge approach is not inferior to trochanteric flip osteotomy for the treatment of Pipkin type IV FH fractures.

## Materials and methods

### Patients

Between 2011 and 2020, 68 patients with Pipkin type IV FH fractures underwent surgical treatment at a referral hospital and level 1 trauma center in North Taiwan. The inclusion criteria were Pipkin type IV fractures, open reduction and internal fixation of the FH and acetabular posterior wall (PW) through a modified Gibson skin incision, and complete radiographic and clinical follow-up for a minimum of 1 year. Patients who were aged < 18 years or had simultaneous fractures of the FH and neck were excluded from the study. Eighteen patients treated with Kocher-Langenbeck incisions to fix the acetabular PW only were excluded from this study; therefore, data from 40 patients were available.

**Fig. 1:**
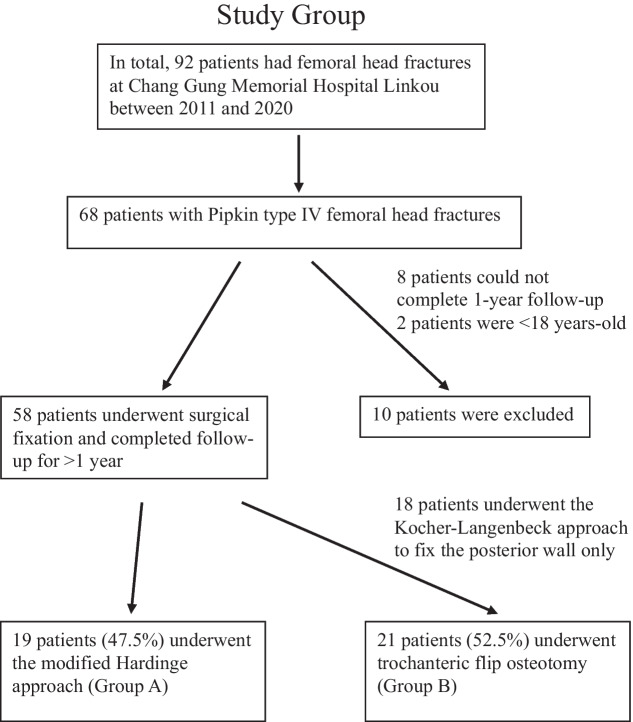
Study flowchart. This image illustrates the study group’s inclusion/exclusion tree

Before July 2017, we used the modified Hardinge approach for managing Pipkin type IV fractures. Subsequently, we have adopted trochanteric flip osteotomies for the management of these fractures. Finally, 19 and 21 patients were treated using the modified Hardinge approach (Group A) and trochanteric flip approach (Group B), respectively. A flowchart of the study is shown in Fig. 1.

The surgical indication for FH fractures was incongruent FH after closed reduction of the dislocated hip joint. Infrafoveal FH fracture with congruent FH on radiography or CT after closed reduction was not fixed, and 18 patients were excluded from this study. Indications for acetabular fractures were displacement > 2 mm or hip joint instability. Fracture displacement was measured using pre-operative radiography and computed tomography (CT). Hip joint instability was assessed using radiography, CT, and examination under anesthesia.

### Surgical procedures

Under general anesthesia, the patient was placed in the lateral decubitus position with the injured hip facing upward. We used a modified Gibson skin incision, as described by Moed in 2010 [19].

For Group A, the modified Hardinge approach was performed as described in a previous study [18]. Briefly, we released the muscle–tendon junction of the gluteus medius at the point of insertion of the greater trochanter. We then cut the gluteus minimus 1 cm medial to its edge; a Z-shaped capsulotomy was performed to release the anterior capsule, and the medial femoral circumflex artery was carefully avoided. The hip joint was then dislocated, and the fractured fragments of the FH were viewed. One to three 3.5-mm interfragmental screws were used to fix and compress the fracture site. The hip was reduced, and the anterior capsule, gluteus minimus, and gluteus medius were repaired layer-by-layer.

For Group B, the trochanteric flip approach of Ganz et al. [9] was followed without modifications. Trochanteric osteotomy for trochanteric flip with a maximal thickness of approximately 1.5 cm was performed using an oscillating saw along the osteotomy line just anterior to the posterior insertion of the gluteus medius. A Z-shaped capsulotomy was performed to avoid injuring the deep branch of the medial femoral circumflex artery. The fractured fragments of the FH were reduced and stabilized in the same manner as in the modified Hardinge approach. The trochanteric osteotomy was repaired using two 3.5-mm cortical screws.

We then performed hip internal rotation and released the abductors as needed to fix the PW or posterior column (PC) fractures. Through a modified Gibson interval, the injured acetabular PW or PC was reduced, and a prebent Judet reconstruction plate or pelvic-locking plate (Johnson & Johnson, New Brunswick, New Jersey, USA) was placed.

### Follow-up protocol

After the index surgery, plain radiographs of the pelvic anteroposterior view and two Judet oblique views were obtained. Post-operative CT scans were performed to assess the reduction quality. Each patient was allowed toe-touch ambulation for at least 6 weeks, followed by full weight-bearing ambulation for another 6 weeks. Three months after the surgery, the patients were allowed to walk freely without assistance.

Patients were assessed at 1, 3, 6, and 12 months after discharge; thereafter, follow-up was conducted annually. Functional and radiological evaluations were performed and documented during each clinic visit. Functional results were evaluated using the Merle d’Aubigné-Postel score [20]. Peri-operative or post-operative complications, including neurovascular injury, loss of reduction, dislocation, hardware failure or irritation, infection, and venous thromboembolism, were recorded.

### Outcome measures

Four parameters were used to evaluate radiological outcomes: quality of fracture reduction using Matta’s grading system, [21] osteonecrosis of the FH (ONFH) using the Ficat classification, [22] post-traumatic osteoarthritis (PTOA) using the Tönnis classification, [23] and heterotopic ossification (HO) using the Brooker classification [24]. The Merle d’Aubigné-Postel score was used to assess clinical outcomes. Conversion to total hip replacement (THR) was recorded as the primary outcome measure. No THRs were performed immediately after the trauma surgery; all THRs were performed during the post-operative follow-up when indicated.

### Statistical analysis

Numerical data are presented as means ± standard deviation, while categorical data are expressed as absolute frequencies and percentages. A comparison analysis was conducted between the two groups using either the Chi-squared or Fisher’s exact test for categorical data, and the two-tailed Student’s t-test or nonparametric Mann–Whitney U-test for numerical data. Patient survival, censored for THR, was analyzed using Kaplan–Meier curves and log-rank tests.

Two-tailed *P*-values < 0.05 were considered statistically significant. The IBM SPSS Statistics 25 (IBM, Armonk, New York, USA) and STATA 12 software packages (StataCorp LP., College Station, Texas, USA) were used to analyze the data.

### Ethics approval

All procedures involving human participants were performed in accordance with the ethical standards (Institutional Review Board approval number:201900259B0), and the 1964 Helsinki Declaration and its later amendments or comparable ethical standards. The requirement for informed consent was waived by the board owing to the retrospective nature of this study.

## Results

### Patient demographics

The study population comprised 31 males and nine females, with an average age of 29.5 ± 11.4 years at the time of injury. The mean follow-up duration was 31.4 ± 21.3 months. All fractures were associated with posterior hip dislocation. Seven patients had failed closed reduction in the emergency department, while the remaining 33 patients had a time-to-reduction within 3.4 ± 3.1 h. The injuries were secondary to car accidents, motorcycle accidents, and falls from a height in five, 31, and four patients, respectively. Associated injuries were present in 20 patients (six head injuries, four chest injuries, six abdominal injuries, and 16 other fractures), with an average Injury Severity Score of 12.7 ± 6.4.

### Comparison of the two approaches

There were no significant differences in patient characteristics, including age at injury, sex, body mass index, Injury Severity Score, time from injury to surgery, follow-up duration, and length of hospital stay (Table 1).

**Table 1:** Comparison of demographic data between patients with Pipkin type IV femoral head fractures treated with the modified Hardinge approach (Group A) or trochanteric flip osteotomy (Group B)

Variables	Group A (*N* = 19)	Group B (*N* = 21)	*p*
Age at injury, years	26.79 ± 9.82	31.86 ± 12.39	0.163
Male/Female (N, ratio)	16/3	15/6	0.457
BMI	25.20 ± 4.01	25.05 ± 4.56	0.918
ISS	13.00 ± 7.51	12.43 ± 5.36	0.782
Associated injury	12 (63.2)	8 (38.1)	0.113
Mechanism			0.223
Car crash	1 (5.3)	4 (19.0)	
Motorcycle crash	17 (89.5)	14 (66.7)	
Fall from height	1 (5.3)	3 (14.3)	
Hip dislocation	19 (100)	21 (100)	1.000
Failed closed reduction	3 (15.8)	4 (19.0)	1.000
Time to reduction, hours	2.94 ± 2.18	3.82 ± 3.84	0.425
Injury to surgery, days	5.74 ± 2.98	8.55 ± 8.99	0.202
Length of hospital stay, days	12.00 ± 4.93	11.38 ± 6.42	0.736
Follow-up duration, months	36.12 ± 23.80	27.11 ± 18.22	0.184

*BMI* body mass index, *ISS* injury severity score

Of the 19 patients treated using the modified Hardinge approach, five and 14 presented with suprafoveal and infrafoveal fractures, respectively. In the trochanteric flip osteotomy group, six and 15 patients presented with suprafoveal and infrafoveal fractures, respectively. All acetabular fractures involved the PW, and one patient each in group had concomitant PC fractures. There was no difference between the two groups regarding any of the fracture characteristics, including the AO/OTA classification of the FH and acetabular fractures, FH comminution, PW comminution, PW size calculated with Moed’s method [25], PW fracture level, and marginal impaction (Table 2).

**Table 2:** Comparison of fracture characteristics between patients in groups A and B

Variables	Group A (*N* = 19)	Group B (*N* = 21)	*p*
Femoral head fracture characteristics			
Suprafoveal	5 (26.3)	6 (28.6)	0.873
Infrafoveal	14 (73.7)	15 (71.4)	0.873
AO/OTA			
C1	11 (57.9)	11 (52.4)	0.726
C2	8 (42.1)	10 (47.6)	0.726
Femoral head comminution	8 (42.1)	8 (38.1)	0.796
Acetabular fracture characteristics			
PW	18 (94.7)	20 (95.2)	1.000
PC + PW	1 (5.3)	1 (4.8)	1.000
AO/OTA			
A1	18 (94.7)	20 (95.2)	1.000
A2	1 (5.3)	1 (4.8)	1.000
Marginal impaction	7 (36.8)	4 (19.0)	0.208
PW comminution*	8 (42.1)	11 (52.4)	0.516
PW fragment size (Moed’s method)	47.11 ± 31.23	43.00 ± 22.86	0.611
PW fracture that extended into the dome	3 (15.8)	5 (23.8)	0.698

*Comminution was defined as more than two fragments

*PC* posterior column, *PW* posterior wall

The mean operative time was 175.58 ± 54.56 min in Group A and 188.48 ± 74.66 min in Group B (*P* = 0.540). The estimated blood loss was 500.00 ± 315.44 mL in Group B, which was significantly higher than in Group A (246.32 ± 141.35 mL; *P* = 0.002; Table 3). There were no peri-operative complications.

**Table 3:** Comparison of perioperative variables, fracture sequela, and clinical outcomes between patients in groups A and B

Variables	Group A (N = 19)	Group B (N = 21)	*p*
Operative time, mins	175.58 ± 54.56	188.48 ± 74.66	0.540
Estimated blood loss, mL	246.32 ± 141.35	500.00 ± 315.44	0.002†
Fracture reduction, Matta grade I / II (N, ratio)	16/3	18/3	1.000
ONFH	4 (21.1)	5 (23.8)	1.000
PTOA	3 (15.8)	2 (9.5)	0.654
HO	1 (5.3)	1 (4.8)	1.000
Trochanteric union	NA	21 (100)	NA
Conversion to THR	4 (21.1)	3 (14.3)	0.689
Mean time to THA, months	21.38 ± 19.65	20.00 ± 24.76	0.857
THA in one year	2 (10.5)	2 (9.5)	1.000
Merle d’Aubigné-Postel score*	16.11 ± 1.41	16.19 ± 1.17	0.836
Patient request screws removal	NA	2 (9.5)	NA

*The score was recorded 1 year after fracture

^†^*p*-value < 0.05

*ONFH* osteonecrosis of the femoral head, *PTOA* posttraumatic osteoarthritis, *HO* heterotopic ossification, *THR* total hip replacement, *NA* not available

**Fig. 2:**
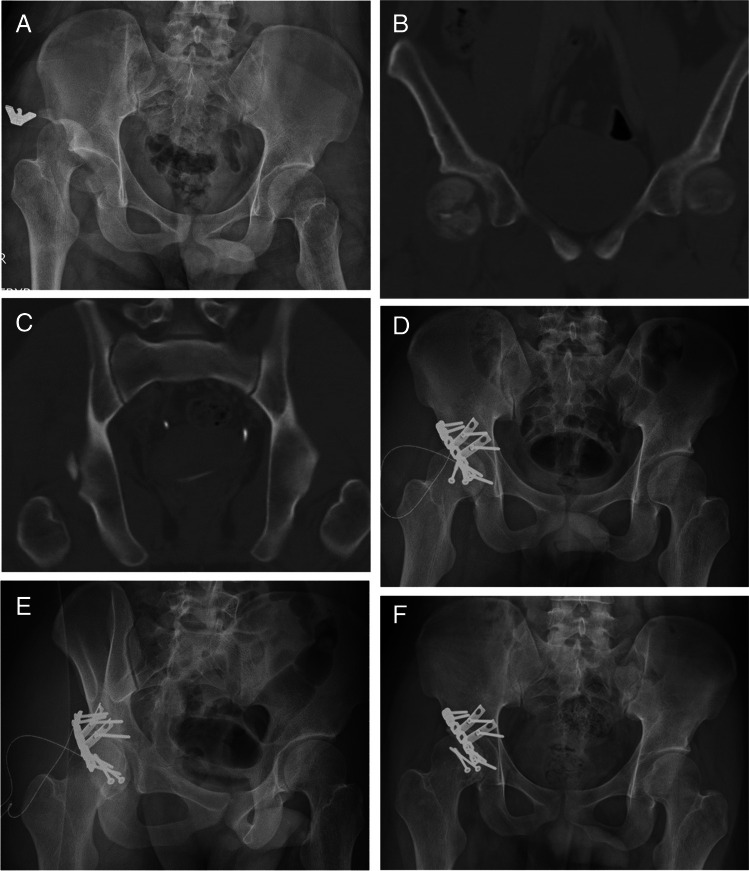
Representative case of an 18-year-old man involved in a motorbike accident. **A** Right hip traumatic fracture-dislocation of an 18-year-old male after a motorbike accident. **B** and **C** CT scan after dislocation reduction showing femoral head and acetabular posterior wall fracture. The patient was operated with the modified Hardinge approach. **D** Post-operative anteroposterior, and **E** obturator oblique radiographs demonstrating congruent hip joint. **F** Radiograph 14 months after the operation showing no signs of osteonecrosis or osteoarthritis. CT, computed tomography

**Fig. 3:**
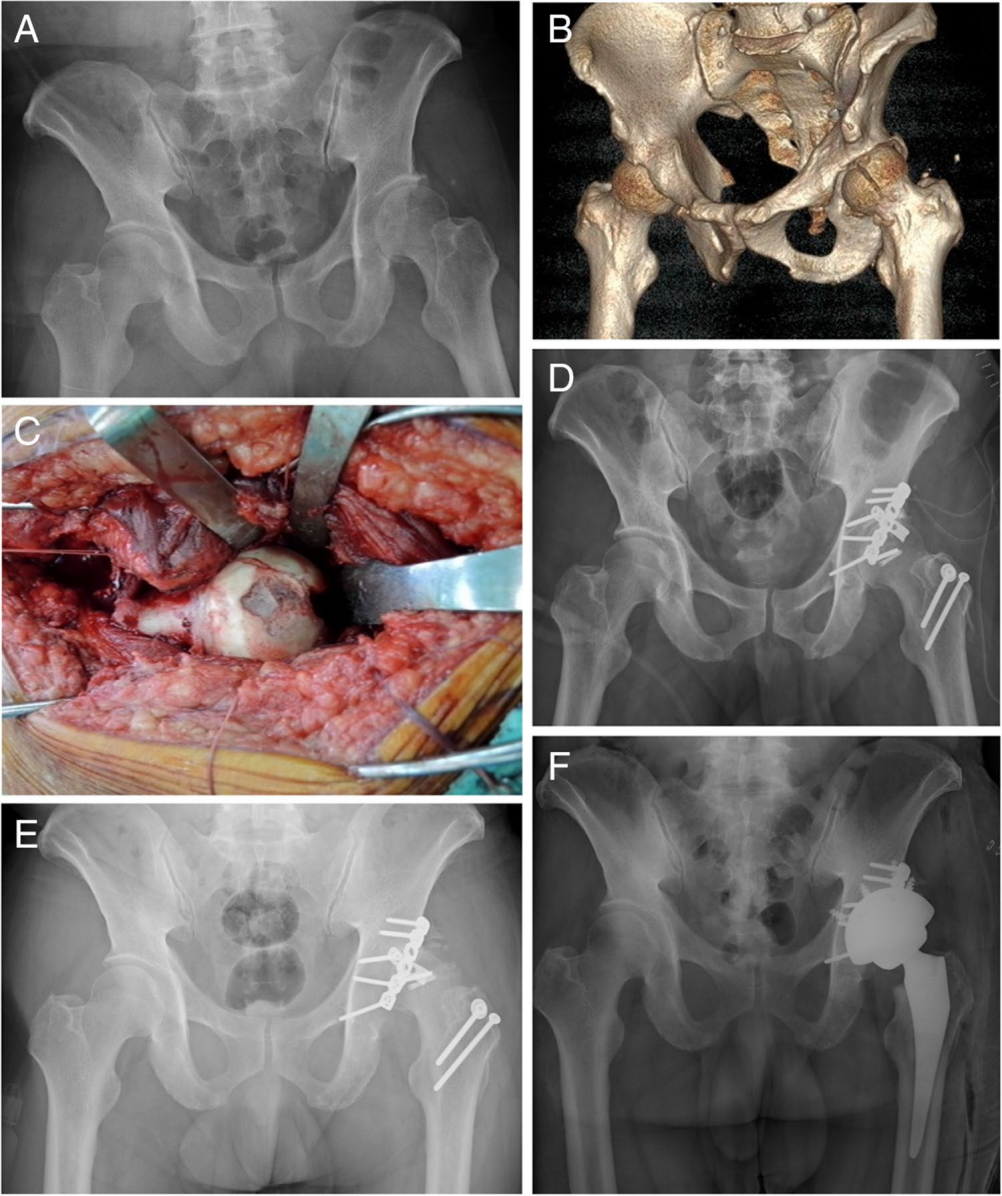
Representative case of a 53-year-old man involved in a motorbike accident. **A** Left hip Pipkin type IV femoral head fracture of a 53-year-old male after a motorbike accident. **B** 3D CT images demonstrating displaced femoral head and acetabular posterior wall fracture. **C** Intra-operative photograph showing femoral head impaction and suprafoveal fracture. **D** Post-operative radiograph after the patient was operated through trochanteric flip osteotomy. **E** Seven months after the index surgery, trochanteric osteotomy healed and left femoral head osteonecrosis was noted. **F** Total hip replacement was performed 11 months after the index surgery. CT, computed tomography, 3D, three dimensional

With regard to radiographic outcomes, the quality of fracture reduction according to Matta’s grading did not differ significantly between the groups (*P* = 1.000), nor did the occurrence of ONFH (Group A vs. Group B, 21.1% vs. 23.8%, *P* = 1.000), incidence of PTOA (15.8% vs. 9.5%, *P* = 0.654), or development of HO (5.3% vs. 4.8%, *P* = 1.000). Radiographs of the cases in each group are shown in Figs. 2 and 3.

Regarding clinical outcomes, the median Merle d’Aubigné-Postel score 1 year postinjury was 16.11 ± 1.41 for Group A and 16.19 ± 1.17 for Group B (*P* = 0.836; Table 3). Regarding post-operative complications, no cases of nerve injury, loss of reduction, hardware failure, infection, or symptomatic venous thromboembolism were detected in either group. One 27-year-old male patient in Group A had recurrent dislocation 11 days after the index surgery. A revision surgery was performed, and the dislocation was resolved. All trochanteric osteotomies healed uneventfully (mean healing time: 16.26 ± 3.82 weeks). Two patients complained of discomfort caused by the trochanteric screws and requested implant removal.

### Primary outcome measure: conversion to total hip replacement

**Fig. 4:**
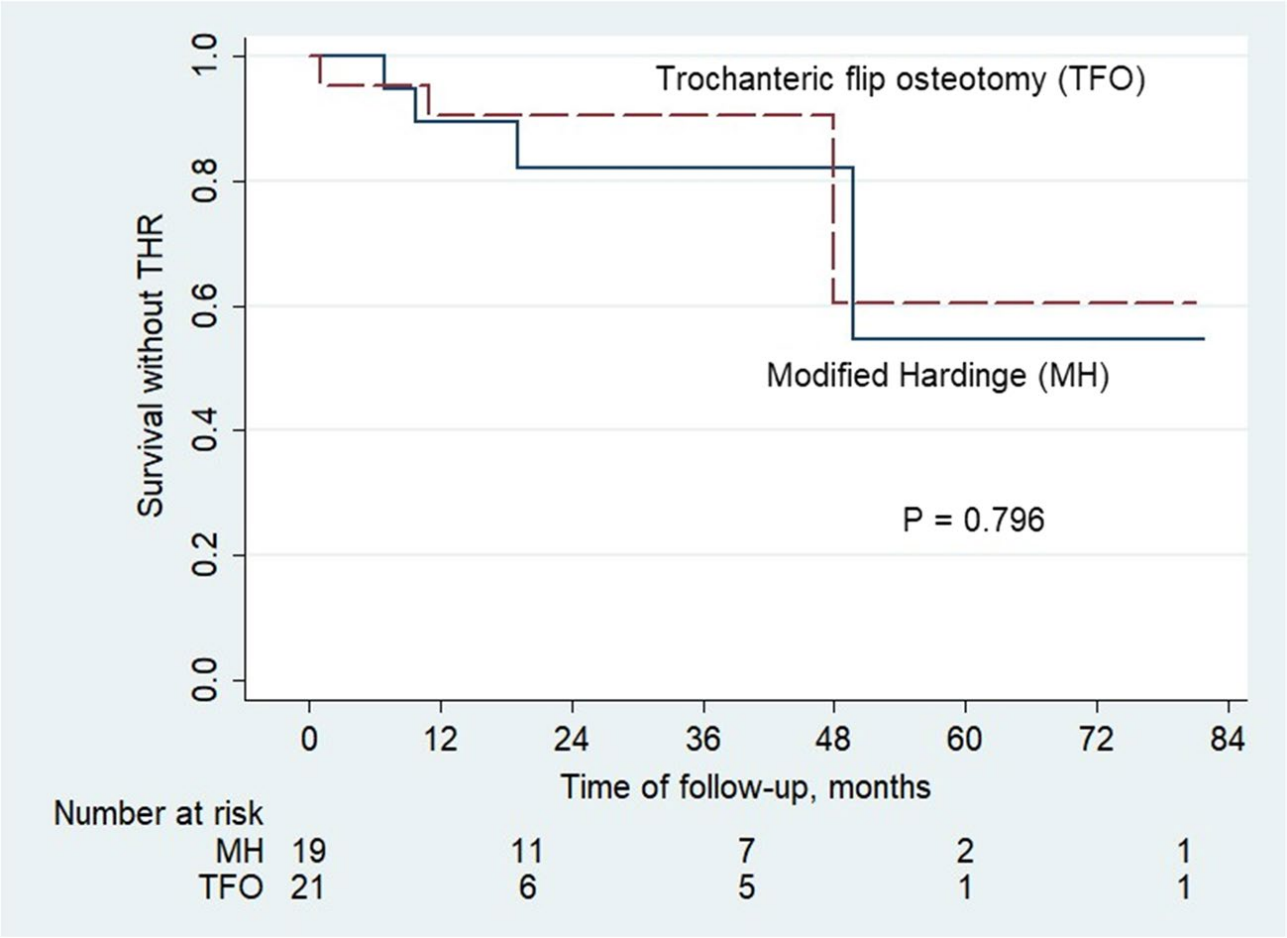
Kaplan–Meier survival curve for patients with Pipkin type IV fractures. Curves of patients treated with the modified Hardinge approach (*n* = 19; blue line) and trochanteric flip osteotomy (*n* = 21; red dashed line) are shown. Total hip replacement was the censored value. Abbreviations: THR, total hip replacement

Seven patients underwent THR during the follow-up period: four (57.1% of THRs) within 12 months, and three 12 months after the initial surgery. Figure 4 shows the Kaplan–Meier survival curve without THR for all patients. In Group A, three patients with Ficat stage IV ONFH and one patient with Tönnis grade III hip joint OA received THR at a mean duration of 21.38 ± 19.65 months after initial surgery. Three patients in Group B had stage IV ONFH during follow-up, and THR was performed 20.00 ± 24.76 months postoperatively. The log-rank test showed no significant difference between the two groups (*P* = 0.796).

Notably, patients with suprafoveal FH fractures exhibited a higher incidence of undergoing THA compared to those with infrafoveal FH fractures during the follow-up period (*P* = 0.011). Among patients with suprafoveal FH fractures, six out of eleven (54.5%) developed ONFH, and five (45.5%) underwent THR. Conversely, in the infrafoveal FH fracture group, only three out of twenty-nine patients (10.3%) were diagnosed with ONFH, and two (6.9%) underwent THR during the follow-up.

## Discussion

Approaches for the management of FH fractures have been well described in the literature; however, relatively few reports exist concerning combined FH and acetabular fractures (Pipkin type IV) owing to the complexity of this condition, and the optimal surgical approach for Pipkin type IV fractures remains under debate [15, 26–29]. The rarity of Pipkin type IV fractures complicates comparative studies. To the best of our knowledge, the present study is the first to compare the different surgical approaches for Pipkin type IV FH fractures.

Several approaches toward surgical hip dislocation have been suggested for the management of FH fractures, including trochanteric flip osteotomy [9, 12]. This approach with surgical dislocation of the hip can achieve accurate reduction and fixation of both FH and acetabular fractures, with a low risk of compromising the FH blood supply [6, 9–11, 26, 30, 31]. However, complications following trochanteric flip osteotomy are frequently observed; including nonunion, trochanter migration, and trochanteric bursitis caused by the prominent implants [9, 15, 16]. Two patients in our series also complained of trochanteric screw prominence and requested screw removal.

In a previous study, a modified Hardinge approach was proposed to prevent these morbidities [18]. In the present study, we further demonstrated that the modified Hardinge approach and trochanteric flip osteotomy could achieve similar radiographic outcomes, including the quality of fracture reduction, ONFH, PTOA, and HO. The clinical outcomes assessed using the Merle d’Aubigné-Postel score and rate of conversion to THR did not differ significantly between the two groups. We also showed that there was no significant difference in the operative time between the two procedures. However, the surgical blood loss in the osteotomy group was twice that in the modified Hardinge group, which was likely due to the bony procedure of the osteotomy.

The risk of ONFH after surgical hip dislocation is concerning. Ganz et al. [9] reported no incidence of osteonecrosis in 213 hips treated with trochanteric flip osteotomy with surgical dislocation of the hip. This finding appears to be significantly different in traumatic cases, where the rate of osteonecrosis in acetabular and FH fractures treated with the trochanteric flip osteotomy method is reported to range from 0–25% [6, 11–13, 25, 29, 30]. The overall incidence of ONFH in our series was 22.5% (940), which is consistent with that reported in the literature. In our procedures, a Z-shaped capsulotomy was carefully performed, and electrocautery was used meticulously to avoid jeopardizing the blood supply to the FH. However, compromised FH blood supply is associated with the surgery itself, as well as the surgical timing and injury pattern [6, 12]. The risk of ONFH after the modified Hardinge approach or trochanteric flip osteotomy requires further studies with larger case series and prospective study designs.

Conversion to THR after a Pipkin type IV FH fracture is considered a treatment failure according to the definitions of many investigators [7, 16]. Some authors have suggested THR as the primary treatment in acute settings, particularly for lesions involving femoral neck fracture in elderly patients [6, 28]. However, insufficient and unstable bone stock render THR extremely difficult to achieve. In this study, we aimed to perform reduction and fixation surgeries in a single attempt. Seven of 40 patients (17.5%) required THR during follow-up; this result is in agreement with the literature, as a THR of 5–33% after Pipkin type IV FH fractures is generally reported [7, 12, 27, 29].

This study has some limitations that should be noted. Firstly, the power of our analysis was insufficient owing to the small number of patients in each group. For a statistical power of 0.8, a sample size of 100 in each group is needed. As Pipkin type IV FH fractures are uncommon, a multicenter study is needed to collect a sufficient number of cases. Secondly, this study was conducted at a single center; therefore, the reproducibility of the procedure is questionable. Given that our institution is one of the largest trauma centers in Taiwan and that surgeons are experienced in treating acetabular and FH fractures, our results may not be reproduced by institutions that do not encounter high volumes of Pipkin type IV fractures. Thirdly, due to the retrospective study design, randomization of patients into one of the two surgical approaches was not feasible. Evaluation of clinical mid- and long-term outcomes based on prospective randomized studies is necessary to ascertain whether the modified Hardinge approach is equivalent to or superior to trochanteric flip osteotomy. Fourthly, ONFH was primarily assessed using plain film imaging following the Ficat classification. Only nine patients (22.5%) underwent magnetic resonance imaging (MRI) within the study period. Early diagnosis of osteonecrosis may be challenging with conventional radiography alone.

In summary, the modified Hardinge approach is reliable for managing FH and posterior acetabular fractures without osteotomy. In this comparative study, there were no significant differences between the approaches regarding radiographic and clinical outcomes. We believe that the modified Hardinge approach may be performed in lieu of trochanteric flip osteotomy to treat Pipkin type IV fractures without the risk of complications associated with trochanteric osteotomy. Nevertheless, further studies with a larger number of patients and longer follow-up periods are required to confirm our conclusions.

## Data Availability

No datasets were generated or analysed during the current study.
